# Maternal Serum Alamandine Levels in Preeclampsia: Association with Clinical Parameters

**DOI:** 10.3390/jcm15218238

**Published:** 2026-10-22

**Authors:** Güneş Topçu, Pınar Yıldız, Seda Şenkardeş Kandemir, Zeynep Gökçe, Gözde Konak, İbrahim Kale, Asiye Uzun

**Affiliations:** 1 Department of Obstetrics and Gynecology, Bahcelievler Medipol University Hospital, Istanbul Medipol University, İstanbul 34197, Türkiye; asiye.uzun@medipol.com.tr; 2 Department of Obstetrics and Gynecology, Academic Hospital, İstanbul 34758, Türkiye; pinar.9530@gmail.com; 3 Department of Internal Medicine, Bagcilar Training and Research Hospital, İstanbul 34200, Türkiye; seda_senkardes@hotmail.com; 4 Department of Obstetrics and Gynecology, Umraniye Training and Research Hospital, İstanbul 34773, Türkiye; dr.zeyneppgokce@gmail.com (Z.G.); dribakale@hotmail.com (İ.K.); 5 Department of Obstetrics and Gynecology, Hekimhan State Hospital, Malatya 44410, Türkiye; drgozdekonak@gmail.com

**Keywords:** alamandine, endothelial dysfunction, preeclampsia, renin–angiotensin system, umbilical artery Doppler

## Abstract

**Objectives:** Preeclampsia is a gestational disorder characterized by endothelial dysfunction and renin-angiotensin system imbalance. Alamandine, a component of its protective pathways, has vasodilatory effects. This study aims to compare maternal serum alamandine levels between preeclamptic and normotensive pregnancies and evaluate their associations with clinical and laboratory parameters. **Methods:** This prospective observational case–control study included 40 women with preeclampsia and 47 normotensive women with singleton pregnancies in the third trimester between June and December 2025. Maternal serum alamandine levels were measured using the ELISA method. Participants’ demographic, clinical, laboratory, umbilical artery Doppler, and perinatal data were prospectively recorded. Alamandine levels were compared between groups; their associations with clinical and laboratory parameters were evaluated using correlation and regression analyses. **Results:** Maternal serum alamandine levels were significantly lower in women with preeclampsia compared to normotensive controls (151.0 ± 51.2 vs. 174.0 ± 39.5 pg/mL; *p* = 0.019). However, the between-group difference was no longer statistically significant after adjustment for maternal age, body mass index, umbilical artery S/D ratio, and aspirin use (B [control − preeclampsia] = 24.76 pg/mL; 95% CI: −3.45 to 52.97; *p* = 0.085). Serum alamandine concentrations showed weak inverse correlations with systolic and diastolic blood pressure. A weak inverse correlation was also observed with the spot urine protein-to-creatinine ratio; however, this association did not remain statistically significant after correction for multiple comparisons. In the multivariate analysis, diastolic blood pressure and the umbilical artery S/D ratio were independently associated with maternal serum alamandine levels. **Conclusions:** Maternal serum alamandine levels were lower in women with preeclampsia in the unadjusted analysis; however, this difference was not statistically significant after expanded multivariable adjustment. Alamandine concentrations showed associations with selected hemodynamic parameters, whereas the relationship with proteinuria remained uncertain. These findings suggest a potential role of circulating alamandine in the vascular alterations associated with preeclampsia; however, its predictive or prognostic value cannot be determined from the present study.

## 1. Introduction

Preeclampsia is a multisystemic disorder that develops after the 20th week of pregnancy and is characterized by hypertension and proteinuria. It poses a serious threat to maternal and fetal health. Although its etiology remains incompletely understood, preeclampsia is associated with severe maternal complications, including myocardial infarction, stroke, acute respiratory distress syndrome, pulmonary edema, renal failure, coagulation disorders, and eclampsia [1,2]. The reported incidence of preeclampsia is approximately 2–8% [1]. Risk factors are previous preeclampsia, chronic hypertension, pregestational diabetes, kidney and autoimmune diseases, and multiple pregnancies. Despite postpartum remission, it is associated with an increased long-term risk of hypertension and cardiovascular disease [3].

Studies have examined various biomarkers, including angiogenic factors, inflammatory cytokines, oxidative stress, and markers of endothelial dysfunction, to facilitate early diagnosis of pre-eclampsia and predict its severity [4]. Although the sFlt-1/PlGF ratio is one of the most valuable predictors in clinical practice, the complex pathophysiology of preeclampsia necessitates the investigation of new biomarkers.

The renin-angiotensin system (RAS) has a key role in maternal vascular adaptation and placental development during normal pregnancy. In preeclampsia, by contrast, activation of the vasoconstrictive ACE/Ang II/AT1R axis and impairment of protective ACE2-related pathways are thought to contribute to the underlying pathophysiology [5,6,7,8].

Alamandine is a bioactive heptapeptide formed by ACE2-mediated hydrolysis of angiotensin A or by decarboxylation of Ang-(1–7), and it displays its effects primarily through the MrgD receptor [9,10,11]. The alamandine/MrgD signaling pathway promotes endothelium-dependent vasodilation and increases NO bioavailability [10,11]. It also has antioxidant, anti-inflammatory, antihypertensive, antifibrotic, and cardiovascular- and renal-protective effects [10,11,12]. Therefore, alamandine, located in the protective arm of the RAS, is considered a potential biomarker in preeclampsia.

This study aims to compare maternal serum alamandine concentrations between pregnant women with preeclampsia and normotensive pregnant women and to evaluate the association between serum alamandine levels and the clinical and laboratory characteristics of preeclampsia.

## 2. Materials and Methods

This prospective, single-center, case-control study was conducted from 1 June to 30 December 2025, at the Department of Obstetrics and Gynecology, Ümraniye Training and Research Hospital, İstanbul, Türkiye. Pregnant women aged 18–45 years with a singleton pregnancy in the third trimester who provided written informed consent were eligible for inclusion. Women younger than 18 or older than 45 years, those with multiple pregnancies or pregnancies conceived through in vitro fertilization, and those with chronic hypertension, pregestational diabetes mellitus, chronic kidney disease, vascular disease, thrombophilia, autoimmune disease, or congenital uterine anomalies were excluded. Women with a history of preeclampsia in a previous pregnancy, concurrent infectious disease during pregnancy, or other obstetric complications were also excluded. Initially, 45 pregnant women with preeclampsia and 47 normotensive pregnant women who met the eligibility criteria were enrolled. The control group comprised pregnant women routinely followed at the same center who had no pre-existing medical conditions or pregnancy-related complications.

A total of 92 pregnant women, including 47 normotensive women and 45 women with preeclampsia, were enrolled between June and December 2025. Five women in the preeclampsia group were subsequently excluded: one because perinatal outcome data were unavailable, two because serum samples were damaged during transport, and two because the corresponding serum measurements did not meet the predefined quality-control criteria. Thus, the final analysis included 87 pregnant women, comprising 47 normotensive women and 40 women with preeclampsia.

The diagnosis of preeclampsia was based on the criteria outlined in the American College of Obstetricians and Gynecologists (ACOG) Practice Bulletin No. 222 [1]. Preeclampsia was defined as proteinuria or target organ impairment in previously normotensive pregnant women, with systolic blood pressure ≥140 mmHg and/or diastolic blood pressure ≥90 mmHg on two measurements taken at least 4 h apart after the 20th week of gestation. Proteinuria was determined as a 24-h urine protein level of ≥300 mg or a spot urine protein-to-creatinine ratio of ≥0.3. Target organ involvement was defined as the presence of at least one of the following: thrombocytopenia (<100,000/µL), a ≥2-fold increase in liver transaminases, renal failure (creatinine >1.1 mg/dL or a doubling from baseline), pulmonary edema, or new-onset neurological or visual symptoms.

Demographic, clinical, laboratory, and obstetric data for all participants were documented. After at least 10 min of rest on the day the blood sample was collected, systolic and diastolic blood pressure and pulse rate were measured. On the same day, the same physician evaluated gestational age, amniotic fluid index, and umbilical artery Doppler parameters (systolic-to-diastolic ratio (S/D), pulsatility index (PI), and resistance index (RI)) by obstetric ultrasound using the same ultrasound device (MyLab™ 6, Esaote Biomedica S.p.A., Genoa, Italy). Laboratory evaluations included maternal serum alamandine levels, complete blood count, alanine aminotransferase (ALT), aspartate aminotransferase (AST), lactate dehydrogenase (LDH), urea, creatinine, prothrombin time (PT), activated partial thromboplastin time (aPTT), and spot urine protein-to-creatinine ratio. Perinatal outcomes assessed included gestational age at delivery, birth weight, newborn sex, 1- and 5-min Apgar scores, live birth, and neonatal intensive care unit (NICU) admission.

Blood samples for alamandine measurement were collected regardless of fasting status and before the initiation of any antihypertensive or magnesium sulfate treatment in women with preeclampsia. From each participant, 5 mL of venous blood was obtained from the antecubital vein into serum separation tubes. Samples were collected after diagnosis in the preeclampsia group and during routine antenatal follow-up in the control group, regardless of fasting status. The serum specimens were centrifuged at 1000 rpm for 15 min after clotting and stored at −80 °C until analysis. Samples were dissolved only once before analysis, and to minimize analytical variability, all samples were analyzed in the same batch. Serum alamandine levels were measured using a commercial sandwich ELISA kit (Human Alamandine ELISA Kit; A.B.T. Laboratory Industry, Ankara, Türkiye; Cat. No.: ABT0113Hu) in accordance with the manufacturer’s instructions. The kit’s measurement range was 3.13–200 pg/mL, its analytical sensitivity was 1.74 pg/mL, and the manufacturer-reported coefficient of variation was <10%. According to the manufacturer, the assay does not show significant cross-reactivity with homologous peptide analogues. The manufacturer also notes that quantitative immunoassay measurements may yield higher absolute concentrations than mass-spectrometry-based methods; therefore, values obtained using these different analytical platforms should not be considered directly interchangeable. Measurements were taken in duplicate, and optical density was measured at 450 nm using a microplate reader. Samples were coded to protect patient identities and study group information, and laboratory personnel performed the analyses blinded to group assignments.

The study’s primary endpoint was a comparison of alamandine concentrations in maternal serum between pregnant women with preeclampsia and those with normotensive pregnancies. The secondary endpoints evaluated the relationship between maternal serum alamandine concentrations and the clinical and laboratory characteristics of preeclampsia.

### Statistical Analysis

Because no previous study has reported serum alamandine levels in patients with preeclampsia or hypertension, an empirical estimate of the effect size could not be obtained. Therefore, a power analysis was performed using G*Power (version 3.1.7) to determine the required sample size. The statistical power of the study was expressed as 1 − β, where β represents the probability of a Type II error. Based on Cohen’s conventions for effect sizes, the calculation assumed a large effect size (d = 0.8) for the comparison between two independent groups. Accordingly, with a significance level of α = 0.05 and 80% power, a minimum of 26 participants per group was required.

Statistical analyses were carried out using JAMOVI (Version 2.6.44). Normality of continuous variables was assessed using the Kolmogorov–Smirnov test. Groups were compared using the independent-samples *t*-test, Welch’s *t*-test, or the Mann–Whitney U test, as appropriate according to distributional and variance characteristics. Relationships between variables were examined using Bayesian Pearson, Bayesian Kendall’s tau, and Spearman correlation analyses. In Bayesian analyses, the strength of evidence was assessed using BF_10_; values of 1–3 were classified as anecdotal, 3–10 as moderate, 10–30 as strong, 30–100 as very strong, and >100 as extremely strong evidence. A BF_10_ value <1 indicated no support for the alternative hypothesis. Multivariable general linear models with HC3 heteroscedasticity-consistent standard errors were used to examine the associations of serum alamandine concentrations with study group, systolic blood pressure, and diastolic blood pressure. Statistical significance was defined as *p* < 0.05.

## 3. Results

Compared with the normotensive group, women with preeclampsia had higher maternal age, BMI, systolic and diastolic blood pressure, and umbilical artery PI (*p* < 0.001, *p* < 0.001, *p* < 0.001, *p* < 0.001, and *p* = 0.026, respectively). No significant differences were detected in other clinical and Doppler parameters (*p* > 0.05). In the preeclampsia group, gestational age at delivery, birth weight, and 1- and 5-min Apgar scores were lower, whereas NICU admission rates were higher (*p* < 0.001 for all comparisons) (Table 1).

In the preeclampsia group, white blood cell (WBC) count and spot urine protein-to-creatinine ratio were significantly higher than in the control group (*p* < 0.001 and *p* = 0.002, respectively), while LDH and creatinine levels were lower (*p* = 0.007 for both). No significant differences were observed in other laboratory parameters (*p* > 0.05). Maternal serum alamandine levels were significantly lower in the preeclampsia group compared to the control group (151.0 ± 51.2 vs. 174.0 ± 39.5 pg/mL; *p* = 0.019). The mean difference was 23.2 pg/mL (95% CI: 3.8–42.6), and Cohen’s d was 0.51 (95% CI: 0.08–0.94) (Table 2). After adjustment for maternal age and BMI, preeclampsia remained associated with lower alamandine levels (B = −29.66 pg/mL, 95% CI: −58.16 to −1.16; *p* = 0.042).

The relationship between alamandine levels and selected maternal, obstetric, and neonatal variables was evaluated using Bayesian correlation analysis. Results of the analysis revealed a moderate association between alamandine levels and the study group, umbilical artery S/D ratio, and mode of delivery. Conversely, only anecdotal evidence of an association was found with aspirin use and NICU admission. We found no evidence of an association between maternal age, body mass index, and alamandine levels (Table 3).

The relationship between serum alamandine levels and clinical and laboratory parameters was assessed by Spearman’s correlation analysis. A significant negative correlation was found between serum alamandine levels and systolic (r = −0.283, *p* < 0.01) and diastolic (−0.317, *p* < 0.01) blood pressures; however, no significant correlation was found regarding gestational age. Also, a weak negative correlation was observed with the spot urine protein-to-creatinine ratio; however, it did not remain statistically significant after Holm correction (adjusted *p* = 0.220). We observed no significant correlation with other laboratory parameters as well (Table 4).

Three separate multivariable linear regression models with HC3 robust standard errors were fitted to examine the associations of maternal serum alamandine concentrations with study group, systolic blood pressure (SBP), and diastolic blood pressure (DBP). Each model included maternal age, body mass index, umbilical artery S/D ratio, and aspirin use as covariates (n = 87). In the group model, the adjusted difference between controls and women with preeclampsia was not statistically significant (B [control − preeclampsia] = 24.76 pg/mL, 95% CI: −3.45 to 52.97; *p* = 0.085). In the separate blood pressure models, SBP showed a negative but non-significant association with alamandine concentrations (B = −0.51 pg/mL per mmHg, 95% CI: −1.06 to 0.04; *p* = 0.068), whereas DBP showed a significant inverse association at the nominal significance level (B = −0.86 pg/mL per mmHg, 95% CI: −1.62 to −0.10; *p* = 0.027). These blood pressure models were fitted to the pooled sample without adjustment for study group. The umbilical artery S/D ratio was positively associated with alamandine concentrations across all three models (all *p* ≤ 0.003). Sensitivity analyses excluding the three aspirin users yielded similar results (n = 84). The robust omnibus tests for these models are presented in Supplementary Table S1.

Additional sensitivity analyses were performed by including LDH and serum creatinine as covariates. After this adjustment, the between-group difference remained statistically non-significant (*p* = 0.1051), while the inverse association between DBP and alamandine concentrations remained significant at the nominal level (*p* = 0.0325). The association with SBP did not reach statistical significance (*p* = 0.0539). Similar findings were obtained after excluding aspirin users (n = 84). The results of these additional sensitivity analyses are presented in Supplementary Table S2.

## 4. Discussion

In this study, maternal serum alamandine levels were found to be significantly lower in pregnant women with preeclampsia. Alamandine concentrations showed weak inverse correlations with systolic and diastolic blood pressure, whereas the association with proteinuria did not remain statistically significant after correction for multiple comparisons. In the multivariable analysis, diastolic blood pressure and the umbilical artery S/D ratio were independently associated with alamandine levels.

First described in 2013 as a novel component of the RAS, alamandine has subsequently emerged as a potentially important bioactive peptide within the RAS [13]. Alamandine is a peptide in the protective axis of RAS that primarily interacts with the MrgD receptor [14]. Alamandine/MrgD signaling has been reported to increase nitric oxide production and vasorelaxation, and to exert antihypertensive, anti-inflammatory, and antioxidant effects [15,16]. Although the effects of alamandine in humans remain incompletely understood, evidence regarding its cardiovascular effects and potential role in hypertension has been derived predominantly from animal models [11,17]. Prolonged alamandine administration reduced both systolic and diastolic blood pressure and attenuated cardiac hypertrophy and fibrosis in a rat model of renovascular hypertension [17]. Similarly, alamandine administration reduced blood pressure and improved vascular responses in experimental Ang II-induced hypertension [11]. These findings support the concept that alamandine may participate in counter-regulatory mechanisms opposing the vasoconstrictor and pro-remodeling effects of the classical RAS.

A recent scoping review by Flor et al. comprehensively evaluated the biological actions of alamandine and identified 25 eligible experimental studies, with the cardiovascular system being the most extensively investigated area. The available evidence suggests that alamandine exerts potentially beneficial cardiovascular effects, including vasodilatory, antihypertensive, anti-inflammatory, antifibrotic, and anti-apoptotic actions, which appear to be mediated predominantly through the nitric oxide and MAPK-related signaling pathways. Importantly, the authors emphasized that the current evidence is predominantly experimental, underscoring the limited understanding of alamandine’s physiological and pathophysiological significance in humans [16]. The low alamandine levels measured in this study in the preeclampsia group may reflect reduced protective RAS activity associated with increased vascular resistance, endothelial dysfunction, and oxidative stress. Such a reduction could theoretically contribute to an imbalance between vasoconstrictive and vasodilatory mechanisms, thereby favoring increased vascular resistance and endothelial dysfunction.

This current study identified some associations between maternal serum alamandine levels and clinical and hemodynamic indicators of preeclampsia. Alamandine levels showed weak negative correlations with systolic and diastolic blood pressure. Although these associations were small, their direction is consistent with alamandine’s reported vasodilatory and antihypertensive effects [14,18]. The correlation with the spot urine protein-to-creatinine ratio was weak and did not remain statistically significant after Holm correction. Therefore, this finding should be interpreted cautiously and does not support a definitive relationship between circulating alamandine and proteinuria.

Serum alamandine concentrations were positively associated with the umbilical artery S/D ratio. However, the S/D ratio itself did not differ significantly between the preeclampsia and control groups. Therefore, this finding does not support a simple inverse relationship between circulating alamandine concentrations and impaired placental perfusion. Rather, the observed association should be interpreted as exploratory and may reflect complex maternal–fetoplacental hemodynamic interactions rather than a direct relationship with placental vascular resistance. Moreover, circulating alamandine concentrations cannot be assumed to reflect placental alamandine production or local placental RAS activity directly. Further studies assessing alamandine and MrgD expression in placental tissue, together with maternal circulating concentrations, are therefore warranted.

The relationship between alamandine and preeclampsia may be particularly relevant in the context of the altered RAS observed in this disorder. Preeclampsia is characterized by increased vascular sensitivity to angiotensin II, endothelial dysfunction, and abnormal regulation of the RAS [6,19]. The protective arm of the RAS, including ACE2-derived peptides such as angiotensin-(1–7) and alamandine, may counterbalance some of the vasoconstrictive and pro-inflammatory effects of the classical Ang II/AT1 receptor axis [20,21]. Given the vasodilatory and potentially antihypertensive effects attributed to alamandine, reduced circulating alamandine levels could potentially represent another component of an impaired counter-regulatory RAS response in preeclampsia. However, this hypothesis remains speculative in humans and requires confirmation through mechanistic and longitudinal studies.

Taken together, our findings suggest that reduced maternal serum alamandine levels are associated with preeclampsia and may be associated with maternal hemodynamic parameters, although the relationships with proteinuria and fetoplacental vascular resistance remain uncertain. To the best of our knowledge, this is the first study to investigate maternal serum alamandine levels in women with preeclampsia, providing novel human data on a peptide whose biological effects have previously been investigated predominantly in experimental models. The simultaneous assessment of serum alamandine levels with maternal blood pressure, proteinuria, and umbilical artery Doppler parameters represents an additional strength of our study, as it allowed us to explore the potential relationship between the alamandine pathway and both maternal and fetoplacental hemodynamics.

Several limitations of this study should be acknowledged. First, the single-center design and relatively small sample size may limit the generalizability of the findings. In addition, alamandine levels were measured at a single time point, precluding assessment of longitudinal changes during pregnancy. Because serum samples obtained before the development of preeclampsia were not available, it could not be determined whether lower alamandine levels precede preeclampsia or result from the disease. Similarly, postpartum alamandine levels were not assessed, preventing evaluation of whether the observed reduction persists after delivery or is reversible following resolution of pregnancy. The absence of simultaneous measurements of placental alamandine/MrgD expression and other RAS components also limits the ability to elucidate the mechanisms underlying the observed reduction in maternal serum alamandine levels. Furthermore, the a priori sample size calculation assumed a larger effect size (Cohen’s d = 0.80) than the observed between-group effect (d = 0.51), suggesting limited statistical power to detect smaller associations. Although the unadjusted between-group difference was statistically significant, it did not remain significant in the expanded multivariable model. Residual confounding cannot be excluded, and these findings should therefore be interpreted as exploratory.

Another limitation of the study is that a commercial ELISA kit was used to measure serum alamandin concentrations. According to the manufacturer, immunoassay-based measurements may yield higher absolute concentrations than mass spectrometry-based methods. However, this difference has not been independently verified in the present study. Therefore, absolute alamandin concentrations should be interpreted with caution, and direct comparison of results obtained using different analytical methods may not be appropriate.

## 5. Conclusions

This study demonstrated that maternal serum alamandine levels were lower in women with preeclampsia than in normotensive pregnant women and identified associations with selected hemodynamic parameters. These findings suggest a potential relationship between circulating alamandine and the vascular alterations of preeclampsia. However, because alamandine was measured only after disease onset and at a single time point, no conclusions regarding its predictive or prognostic value can be drawn. Larger prospective longitudinal studies with serial measurements beginning before the onset of preeclampsia are needed to clarify its potential clinical relevance.

## Figures and Tables

**Table 1:** Demographic, Clinical, Obstetric, and Perinatal Characteristics of the Preeclampsia and Control Groups.

Variable	Preeclampsia Group (n = 40)	Control Group (n = 47)	*p*-Value
Maternal age (years)	29 (26–34)	25 (21–32)	<0.001
Gravidity	2 (1–3)	2 (1–3)	0.236
Parity	1 (0–1)	1 (0–1)	0.318
BMI (kg/m^2^)	31.3 ± 3.8	27.8 ± 2.6	<0.001
Aspirin use, n (%)	3 (7.5)	0 (0.0)	0.093
Gestational week at blood sampling	35.5 ± 3.2	36.1 ± 3.1	0.359
Gestational week at ultrasound examination	35 (34–37)	35 (34–38)	0.394
Systolic blood pressure (mmHg)	150(148–160)	110 (100–110)	<0.001
Diastolic blood pressure (mmHg)	98 (90–100)	70 (60–70)	<0.001
Umbilical artery S/D ratio	2.27 ± 0.69	2.10 ± 0.51	0.207
Umbilical artery PI	0.98 ± 0.21	0.89 ± 0.15	0.026
Umbilical artery RI	0.61 ± 0.10	0.61 ± 0.08	0.785
Maternal heart rate (beats/min)	80.9 ± 9.23	79.5 ± 7.96	0.472
Gestational week at delivery	37 (36–38)	39 (38–40)	<0.001
Apgar score at 1 min	8 (7–8)	8 (8–9)	<0.001
Apgar score at 5 min	9 (8–9)	10 (9–10)	<0.001
Birth weight (g)	2750 ± 680	3300 ± 580	<0.001
NICU admission, n (%)	18 (45.0)	5 (10.6)	<0.001

Data are presented as mean ± standard deviation, median (interquartile range), or n (%), as appropriate. BMI, body mass index; NICU, neonatal intensive care unit; PI, pulsatility index; RI, resistance index; systolic/diastolic ratio.

**Table 2:** Laboratory Findings in the Preeclampsia and Control Groups.

Laboratory Parameter	Preeclampsia Group (n = 40)	Control Group (n = 47)	*p*-Value
Hemoglobin (g/dL)	11.4 ± 1.3	10.9 ± 1.8	0.218
White blood cell count (×10^3^/µL)	10.8 (9.3–12.3)	7.8 (6.8–8.7)	<0.001
Platelet count (×10^3^/µL)	236 ± 86	269 ± 73	0.051
ALT (U/L)	11.0 (9.0–15.3)	10.0 (7.0–13.5)	0.413
AST (U/L)	16.5 (13.0–20.3)	18.0 (17.0–28.0)	0.735
LDH (U/L)	277 ± 96	333 ± 91	0.007
Urea (mg/dL)	18.5 ± 6.9	20.6 ± 5.7	0.136
Creatinine (mg/dL)	0.65 ± 0.15	0.77 ± 0.26	0.007
Spot urine protein/creatinine ratio *	0.58 (0.33–1.15)	0.10 (0.10–0.17)	0.002
Alamandine (pg/mL)	151.0 ± 51.2	174.0 ± 39.5	0.019

Data are presented as mean ± standard deviation or median (interquartile range), as appropriate. ALT, alanine aminotransferase; AST, aspartate aminotransferase; LDH, lactate dehydrogenase. * Values of the spot urine protein-to-creatinine ratio below the laboratory reporting threshold of 0.10 were reported as 0.10.

**Table 3:** Bayesian Correlations Between Alamandine Levels and Selected Maternal, Obstetric, and Neonatal Variables.

Variable	Correlation Coefficient	BF_10_
Maternal age	−0.097	0.198
Body mass index	0.003	0.134
Umbilical artery S/D ratio	0.279	3.841
Study group (preeclampsia vs. control)	0.203	6.487
Aspirin use	−0.165	1.743
Mode of delivery	−0.198	5.367
NICU admission	−0.178	2.628

Abbreviations: BF_10_, Bayes factor in favor of the alternative hypothesis; S/D, systolic/diastolic ratio; NICU, neonatal intensive care unit. Note: Correlations were assessed using Bayesian Pearson or Bayesian Kendall’s tau correlation analysis, as appropriate. BF_10_ values >1 indicate evidence in favor of an association, whereas BF_10_ values < 1 indicate evidence in favor of the null hypothesis. Coding of categorical variables: preeclampsia status: preeclampsia = 1, control = 2; mode of delivery: vaginal delivery = 1, cesarean delivery = 2.

**Table 4:** Correlations Between Maternal Serum Alamandine Concentrations and Laboratory Parameters.

Variable	Spearman’s ρ	*p*	Holm-Adjusted *p*
Spot urine protein/creatinine ratio	−0.247 *	0.022	0.220
White blood cell count	−0.211	0.051	0.459
Hemoglobin level	0.069	0.527	1
Platelet count	0.068	0.529	1
Alanine aminotransferase	−0.056	0.604	1
Aspartate aminotransferase	−0.021	0.845	1
Lactate dehydrogenase	0.124	0.252	1
Urea	−0.028	0.799	1
Creatinine	−0.024	0.827	1
Gestational age at delivery	0.120	0.270	1

Correlations were assessed using Spearman’s rank correlation test. *p-values* were adjusted for multiple comparisons using the Holm–Bonferroni method. Statistical significance was defined as *p* < 0.05. * Indicates statistical significance before adjustment for multiple comparisons.

## Data Availability

The datasets generated and/or analyzed during the current study are available from the corresponding author on reasonable request.

## Supplementary Materials

The following supporting information can be downloaded at: https://www.mdpi.com/article/10.3390/jcm15218238/s1, Table S1: Robust omnibus and term-level tests for multivariable linear regression models in the full sample and after excluding aspirin users; Table S2: Sensitivity analyses with additional adjustment for lactate dehydrogenase and creatinine: robust omnibus and term-level tests.

## Abbreviations

The following abbreviations are used in this manuscript:

ACOG	American College of Obstetricians and Gynecologists
ACE	Angiotensin-converting enzyme
ALT	Alanine aminotransferase
Ang II	Angiotensin II
aPTT	Activated partial thromboplastin time
AST	Aspartate aminotransferase
AT1R	Angiotensin II type 1 receptor
BF_10_	Bayes factor in favor of the alternative hypothesis
BMI	Body mass index
CI	Confidence interval
ELISA	Enzyme-linked immunosorbent assay
LDH	Lactate dehydrogenase
MAPK	Mitogen-activated protein kinase
MrgD	Mas-related G protein-coupled receptor D
NICU	Neonatal intensive care unit
NO	Nitric oxide
PI	Pulsatility index
PlGF	Placental growth factor
PT	Prothrombin time
RAS	Renin–angiotensin system
RI	Resistance index
S/D	Systolic-to-diastolic ratio
SFlt-1	Soluble fms-like tyrosine kinase-1
STROBE	Strengthening the Reporting of Observational Studies in Epidemiology
WBC	White blood cell
