# Supplementary material for: Maternal Serum Alamandine Levels in Preeclampsia: Association with Clinical Parameters
Source: J Clin Med. 2026 Oct 22;15(21):8238. doi: 10.3390/jcm15218238 (PMC13764571; doi:10.3390/jcm15218238)
Supplement: Supplementary file 1 [file jcm-15-08238-s001.zip › jcm-4586105-supplementary.pdf]

Supplementary Table S1. Robust omnibus and term-level tests for multivariable linear regression models in the full sample and after excluding aspirin users.

Outcome: serum alamandine concentration (pg/mL). Each column represents a separate model, with group status, SBP, or DBP as the predictor of interest.

Term	df	Full sample (N = 87)			Excluding aspirin users (N = 84)		
		Group model	SBP model	DBP model	Group model	SBP model	DBP model
**Overall model**	5 / 4	4.4250 (0.0013)	4.6680 (0.0009)	4.7320 (0.0008)	4.4720 (0.0026)	4.7530 (0.0017)	4.8500 (0.0015)
Age	1	0.0890 (0.7656)	0.1270 (0.7221)	0.0940 (0.7604)	0.1450 (0.7040)	0.1860 (0.6672)	0.1320 (0.7175)
BMI	1	0.0000 (0.9838)	0.0350 (0.8524)	0.0040 (0.9520)	0.0070 (0.9317)	0.0130 (0.9086)	0.0000 (0.9902)
S/D	1	11.1340 (0.0013)	10.1160 (0.0021)	10.8000 (0.0015)	11.0270 (0.0014)	9.9440 (0.0023)	10.3360 (0.0019)
Aspirin use	1	3.2260 (0.0762)	3.5410 (0.0635)	3.0980 (0.0822)	—	—	—
**Predictor of interest (group / SBP / DBP)**	1	3.0490 (0.0846)	3.4220 (0.0680)	5.0420 (0.0275)	3.0620 (0.0840)	3.3850 (0.0696)	5.0920 (0.0268)
**Adjusted R^2**	—	0.166	0.176	0.178	0.143	0.153	0.157

Values are F statistics (p values), except adjusted R^2. The df column gives numerator degrees of freedom for the overall-model F test (full sample / aspirin-excluded sample) and one numerator degree of freedom for individual predictors. SBP, systolic blood pressure; DBP, diastolic blood pressure; BMI, body mass index; S/D, systolic/diastolic Doppler ratio; LDH, lactate dehydrogenase. Aspirin-use terms were not fitted in the subset with aspirin users excluded. Reported p values are unadjusted for multiplicity (no Holm correction).

Supplementary Table S2. Sensitivity analyses with additional adjustment for lactate dehydrogenase and creatinine: robust omnibus and term-level tests.

Outcome: serum alamandine concentration (pg/mL). Each column represents a separate model, with group status, SBP, or DBP as the predictor of interest.

Term	df	Full sample (N = 87)			Excluding aspirin users (N = 84)		
		Group model	SBP model	DBP model	Group model	SBP model	DBP model
**Overall model**	7 / 6	3.3730 (0.0034)	3.7350 (0.0015)	3.6520 (0.0018)	3.2650 (0.0065)	3.6620 (0.0030)	3.5570 (0.0037)
Age	1	0.2280 (0.6344)	0.3930 (0.5327)	0.2760 (0.6011)	0.3570 (0.5517)	0.5460 (0.4624)	0.3730 (0.5433)
BMI	1	0.0150 (0.9017)	0.0200 (0.8892)	0.0010 (0.9693)	0.0420 (0.8387)	0.0020 (0.9663)	0.0140 (0.9076)
S/D	1	11.0270 (0.0014)	10.5460 (0.0017)	11.1250 (0.0013)	11.1700 (0.0013)	10.5980 (0.0017)	10.7930 (0.0015)
Aspirin use	1	3.1740 (0.0786)	3.3830 (0.0696)	3.2530 (0.0751)	—	—	—
LDH	1	1.0420 (0.3106)	1.8050 (0.1829)	1.4220 (0.2367)	0.9650 (0.3290)	1.7200 (0.1936)	1.3030 (0.2572)
Creatinine	1	0.6300 (0.4299)	0.7890 (0.3772)	0.6140 (0.4358)	0.9460 (0.3337)	1.1410 (0.2888)	0.8800 (0.3511)
**Predictor of interest (group / SBP / DBP)**	1	2.6870 (0.1051)	3.8300 (0.0539)	4.7380 (0.0325)	2.8630 (0.0947)	3.9130 (0.0515)	4.8040 (0.0314)
**Adjusted R^2**	—	0.162	0.182	0.178	0.141	0.161	0.156

Values are F statistics (p values), except adjusted R^2. The df column gives numerator degrees of freedom for the overall-model F test (full sample / aspirin-excluded sample) and one numerator degree of freedom for individual predictors. SBP, systolic blood pressure; DBP, diastolic blood pressure; BMI, body mass index; S/D, systolic/diastolic Doppler ratio; LDH, lactate dehydrogenase. Aspirin-use terms were not fitted in the subset with aspirin users excluded. Reported p values are unadjusted for multiplicity (no Holm correction).
